# Synthetic Generation of Passive Infrared Motion Sensor Data Using a Game Engine

**DOI:** 10.3390/s21238078

**Published:** 2021-12-02

**Authors:** Petter Stefansson, Fredrik Karlsson, Magnus Persson, Carl Magnus Olsson

**Affiliations:** 1 Internet of Things and People Research Center, Department of Computer Science and Media Technology, Malmö University, 211 19 Malmö, Sweden; carl.magnus.olsson@mau.se; 2 Sony Network Communications, 223 62 Lund, Sweden; fredrik.karlsson@sony.com (F.K.); magnus.persson@sony.com (M.P.)

**Keywords:** synthetic data generation, simulation, passive infrared, PIR, motion sensor, occupancy count, people count

## Abstract

Quantifying the number of occupants in an indoor space is useful for a wide variety of applications. Attempts have been made at solving the task using passive infrared (PIR) motion sensor data together with supervised learning methods. Collecting a large labeled dataset containing both PIR motion sensor data and ground truth people count is however time-consuming, often requiring one hour of observation for each hour of data gathered. In this paper, a method is proposed for generating such data synthetically. A simulator is developed in the Unity game engine capable of producing synthetic PIR motion sensor data by detecting simulated occupants. The accuracy of the simulator is tested by replicating a real-world meeting room inside the simulator and conducting an experiment where a set of choreographed movements are performed in the simulated environment as well as the real room. In 34 out of 50 tested situations, the output from the simulated PIR sensors is comparable to the output from the real-world PIR sensors. The developed simulator is also used to study how a PIR sensor’s output changes depending on where in a room a motion is carried out. Through this, the relationship between sensor output and spatial position of a motion is discovered to be highly non-linear, which highlights some of the difficulties associated with mapping PIR data to occupancy count.

## 1. Introduction

Being able to automatically determine the number of occupants in an indoor space at any given moment has potential benefits. For instance, user comfort in a building can be increased by implementing occupancy-controlled ventilation [1], and the energy consumption of the building can be decreased by automatically switching off lights in unoccupied parts of the building [2,3]. In systems related to security, occupancy estimation is often used to detect intrusions [4,5], while occupancy detection in industrial workplaces can be used to prevent injuries by raising an alarm when people are detected in unsafe areas [6]. In the event of a fire, especially in high-rise buildings, firefighters can benefit from real-time room occupancy estimates when planning evacuation and rescue missions [7]. Facility managers can use data of historical room usage to gain insight as to whether rooms and buildings are being utilized as intended or not, thus aiding them in their choice of which room types to prioritize in changes or new developments. Due to the SARS-CoV-2 pandemic, the demand for occupancy monitoring has spread to new areas; businesses across the world that previously had no incentive to quantify occupancy must now in some cases comply with regulations regarding occupancy limits to be allowed to operate.

The task of automating people count estimations has been approached in several studies using different methods. Lempitsky and Zisserman developed a supervised learning method able to count the number of people in surveillance video frames [8]. Oosterhout, Bakkes, and Kröse developed a head detection method capable of counting people from stereo camera data [9]. Wang and Jin proposed ways of estimating indoor occupancy by analyzing the carbon dioxide concentration of the return air of a room’s ventilation system [10]. Depatla and Mostofi showed that it is possible to estimate the number of people in an indoor space by transmitting WiFi signals into a room and measure what signal is received at the opposite side of the room after the signal has propagated through the room [11]. Tsou et al., Leech et al., and Raykov et al. used passive infrared, PIR, sensor data together with supervised learning to estimate occupancy [7,12,13].

Each technical approach when attempting to solve the problem of occupancy estimation has its advantages and disadvantages. Computer vision-based approaches can offer a high degree of accuracy in their estimation [6], but vision-based approaches are sensitive to non-uniformity in lighting conditions as well as perspective and scale issues related to varying distances between people and the camera [14]. Camera monitoring can also be perceived as intrusive and poses privacy concerns [15]. Approaches based on analyzing the air of an indoor space are less intrusive but are also slow to react to changes in people count, as there is an inevitable delay between people entering a room and their exhalation impacting the overall carbon dioxide level of the room’s return air. Infrared sensor approaches rely on thermal radiation emitted from humans. In contrast to conventional vision-based approaches, they, therefore, do not require the space to be uniformly lit, and can even operate in darkness. Nor are they as privacy intrusive as conventional video cameras. In a recent study Groß et al. evaluated and compared 18 different sensor technologies for occupancy detection using a Pugh matrix [6]. Each assessed technology was given a rating across ten differently weighted criteria including cost, ease of installation, privacy, social acceptability, etc. The highest scoring solution in their comparison was a passive infrared matrix sensor with a spatial resolution of 32 × 24 pixels. The simpler, non-spatially resolved, PIR sensor ranked fifth highest. The primary reason PIR sensor-based solutions scored comparatively better than conventional techniques such as video camera-based systems—which ranked at 11th place in the Groß et al. study—is that PIR sensors offer a high degree of privacy and social acceptance combined with a low cost and ease of installation. The predominant drawbacks of the simple PIR sensor are its lack of spatial resolution and its limited count range. As the output of PIR sensors in some cases can be binary, it can be challenging to discriminate between movements from one occupant or multiple occupants.

Many of the articles concerned with automated people count estimation rely on some form of supervised machine learning algorithm [7,8,12,13]. In other words, using a labeled dataset with known people counts for every sensor observation, the authors develop a model that learns from the dataset what the correct mapping is between sensor data (PIR data, images from a camera, etc.) and people count. One of the most widely recognized limitations of supervised machine learning models—especially deep learning models—is that they tend to require large amounts of labeled training data before a robust mapping between input and output is converged upon. Using a large dataset as opposed to a small one is often the most effective way to increase the generalization performance of a deep learning model [16]. In the context of occupancy counting, collecting such a large labeled dataset for training a machine learning model can be problematic. Yordan et al.’s occupancy estimation study [13] collected a labeled people count dataset by stationing a person outside the office meeting room where the data collection took place. The person stationed outside the room would then manually count the number of people occupying the room over time. One clear disadvantage of this approach is that roughly one hour of observational work is required for every collected hour of occupancy count. Collecting a large dataset comprised of thousands of hours of occupancy ground truth data could thus require thousands of hours of effort, making the data collection process both costly and time-consuming, as well as arguably tedious for the person tasked with performing the observations. Furthermore, during the pandemic, many workplaces around the world are either empty or operating at an intentionally decreased capacity. Collecting occupancy data for some sites may therefore not currently be feasible. Hence there is a need for better data collection methods that can reduce the time and cost required to obtain a labeled dataset, ideally without the risk of contributing to the spread of infectious diseases. One approach that could potentially solve the mentioned issues is to perform the data collection synthetically in a computer simulation as opposed to collecting the data in the real world.

Synthetic data generation is the process of artificially generating a dataset without necessarily interacting with the real world, usually by means of collecting data from a computer simulation or an algorithm. The generated dataset may then be used to train a supervised machine learning model. Provided that the synthetically generated data has characteristics sufficiently similar to that of data originating from the real-world rendition of the same process as is being simulated, a supervised learning model can be trained using synthetic data and then later be successfully deployed in an environment where it is given non-synthetic data. Utilizing synthetic data generation to solve machine learning problems has become increasingly popular in recent years [17] and has been used in a multitude of applications such as for autonomous driving [18], semantic segmentation [19], text recognition [20] and obstacle detection for unmanned aerial vehicles [21]. On certain tasks, such as estimating the three-dimensional pose of objects for robotic applications, state-of-the-art real-world results have been obtained by training a deep learning model exclusively on synthetically generated data [22].

One of the major benefits of synthetic data generation is that extreme events which rarely occur in reality can be readily generated in large quantities inside a simulator. Another benefit is that unlike labels derived from manual observations that may be prone to human errors, computer-generated labels have a high probability of not containing any mislabeled samples. The main disadvantage of synthetic data generation is that for some problems it is difficult to recreate an accurate simulation of the events being studied. If the synthetic data obtained from a simulation is not realistic enough, then a supervised model trained on the data will not generalize well to data originating from the real-world version of the same process—a problem often referred to as the reality gap [17,22]. The need for a synthetic dataset is often motivated by high costs or safety concerns associated with generating a real-world dataset. For certain events, it may not even be feasible to collect non-synthetic data, such as when developing collision detection systems for drones, where there are stringent regulatory constraints imposed on the type of flights allowed in urban environments [21]. These concerns apply well within the domain of occupancy estimation, and the possibility of collecting motion sensor data for various indoor occupancies synthetically using a simulator should therefore be investigated.

In this paper, we explore the feasibility of using a game engine to simulate the key components involved in the collection of a labeled dataset for occupancy estimation, namely: rooms, occupants, and motion sensors. We use specifications from the technical data sheet of a real-world passive infrared motion sensor to recreate a simulated representation of the sensor capable of detecting motion inside the simulator. Using a collection of primitive three-dimensional objects we constructed a simple humanoid model to represent an occupant, and created a layout of a real-world meeting room inside the simulator which was equipped with artificial PIR motion sensors.

Using the developed simulator we conduct two studies: Study A and Study B. Study A consists of an experiment designed to verify the level of realism in the synthetic motion sensor data produced by the simulator. To achieve this, motion sensor data was first collected from the real-world meeting room where an occupant of the meeting room carried out a predetermined set of motions that were easily reproducible in the simulator. The motion sensor data collection was then performed synthetically using the simulator by letting the humanoid perform the same predetermined set of motions in the same part of the room as were carried out in real life. One of the findings of Study A is that a relatively minor positional change in a room can cause a large change in the motion sensor’s ability to detect the motion. Study B was therefore designed to investigate the relationship between spatial position in the meeting room and the PIR sensor’s ability to detect motion. To accomplish this, the simulated meeting room was partitioned into a grid. Two objects were then rotated for one minute in each of the separate squares of the grid which enabled the PIR sensors’ sensitivity to be measured as a function of spatial position in the room.

The rest of the paper is structured as follows. In Section 2 we describe how we implemented the PIR motion sensors and the human occupant in the simulator, as well as describe the geometry of the room that is used in the simulations. In Section 3 we define the two experiments that were carried out in the simulator. In Section 4 we present the results of the experiments, which are discussed in Section 5. Lastly, some conclusions are given in Section 6.

## 2. Description of Developed Simulator

The Unity^®^ game engine v.2021.1.12.f1 was used to develop the simulator used to conduct the experiments of this paper. To produce synthetic data the simulator needs to be equipped with three main features: motion sensors, a human whose movements the sensor can detect, and an indoor space that can be occupied. How these three features were implemented in the Unity simulator is described in Section 2.1, Section 2.2 and Section 2.3 respectively. If the described simulator was to be used to collect a labeled dataset containing people count as well as motion sensor data, an occupancy counting feature would also have to be added to the simulator. In Unity, such a feature can easily be implemented for instance by continuously querying how many objects are touching the floor of a room at any given moment. Because the experiments of this paper only include one occupant, the implementation of such a feature will not be described here.

### 2.1. PIR Motion Sensors

All bodies with a temperature above absolute zero emit radiation. The greater the temperature of the body is, the greater the energy content of the emitted radiation is; and the shorter the wavelength is at which the emitted radiation peaks. Human bodies are typically around 37 °C internally and 33.5–36.9 °C on the skin surface [23]. Most surfaces in a climate-controlled building, however, are approximately 20 °C. By measuring the radiation intensity in a room it is therefore in most cases possible to differentiate between human-caused emission of thermal radiation and emissions from walls, floors, furniture, and other inanimate objects. The emitted radiation of objects in the 20–37 °C range peaks in the infrared region of the electromagnetic spectrum. It is therefore common to use infrared sensors when monitoring indoor spaces for activity.

Passive infrared, PIR, sensors are commonly used to detect human movement indoors for a variety of purposes such as for occupancy-controlled lighting [2,3] and intruder detection in alarm systems [4,5]. As a person walks into a PIR sensor’s field of view, it can detect an increase in the influx of infrared radiation, and thus the sensor can classify the event as motion. Similarly, as a person exits a PIR sensor’s field of view, the sensor can detect a return to background levels of radiation and can classify the event as motion. When a person is standing or sitting still in a PIR sensor’s field of view, however, it is often difficult for a PIR sensor to detect the person.

**Figure 1:**
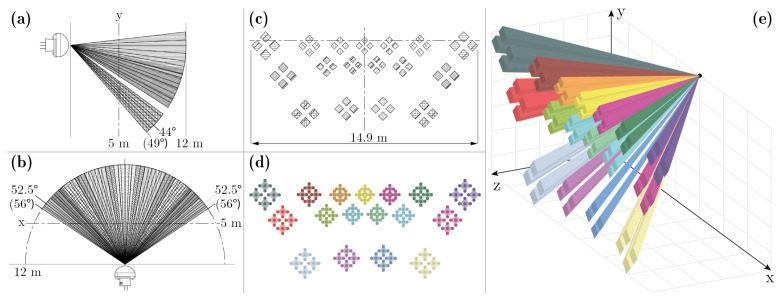
(**a**) side view of the Panasonic EKMC1604111 sensor’s field of view; (**b**) top view of the sensor’s field of view; (**c**) x–y cross-section of the sensor’s beam pattern; (**d**) beam pattern of the synthetic version of the EKMC1604111 sensor; (**e**) perspective view of the synthetic sensor’s beam pattern in 3D space. Subplots (**a**–**c**) are adapted from Panasonic’s technical data-sheet [24].

To better focus the radiation onto the sensor, PIR sensors are often equipped with Fresnel lenses. Fresnel lenses are used to partition a sensor’s field of view into a pattern of different detection zones or beams. The maximum detection distance can vary between beams. The PIR sensors used in the experiments described in this paper are of type Panasonic EKMC16 and are equipped with Panasonic 04111 wall lenses. The product number of the combined use of this sensor and lens is Panasonic EKMC1604111. The field of view and beam pattern of the EKMC1604111 sensor is shown in Figure 1 subplots a–c. The Panasonic 04111 wall lens partitions the field of view into 68 beams formed into 17 clusters containing four detection zones each as shown in Figure 1 subplot c. The detection distance of the beams varies in three steps: the lowermost 4 clusters have a detection distance limit of 3 m, the 6 middlemost clusters have a detection distance limit of 6 m, the 7 uppermost clusters have a detection distance limit of 12 m.

To recreate the functionally of the Panasonic EKMC1604111 sensor in the simulator, angles and measurements from the sensor’s accompanying technical data-sheet [24] were used to construct a mathematical representation of the sensor’s field of view. Since the drawings of the technical data-sheet can be downloaded in a vectorized format, any needed measurements that were not directly annotated in the drawings could accurately be determined by importing the technical data-sheet into AutoCAD and annotating them there.

Instead of assigning temperatures to objects, our developed Unity simulator uses ray casting to detect motion. Ray casting is a computational method where a point source is defined in three-dimensional space along with a direction. The ray defined by the source point and direction is then traced in an attempt to find intersections with other objects in the modeled 3D world. Since ray casting operates along a one-dimensional line, and the beams of the Panasonic PIR sensor are two-dimensional zones projected into 3D space, an infinite number of rays would be required to perfectly cover the zones of the PIR sensor. Since this is not practically possible to simulate, each detection zone of the PIR sensor was instead simplified and represented as five points: one in each corner of the detection zone and one in the center. In total 340 (5 × 4 × 17) ray casts were used in our simulations to model the beam pattern of the PIR sensor. In accordance with Panasonic’s datasheet, the rays were given different maximum projection distances based on the detection distances of the various beams: 3 m for the lower clusters, 6 m for the middle clusters, and 12 m for the upper clusters. The beam pattern of the simulated PIR sensor is shown in Figure 1 subplots d and e. When ray casting in Unity, our simulated sensors were configured to ignore intersections with all objects that were not tagged with the name “Human”. The “Human” tag was only given to the body parts of the humanoid that is described in Section 2.2. Ray cast intersections with the humanoid could thus trigger the PIR sensors, whereas objects such as walls, floor, and furniture could block the sensor’s field of view, but not trigger a motion event in the sensor.

In the PIR sensors in question, motion is defined as any change in activity in any of the detection zones. For instance, if one of the rays in one of the 17 detection zones detects a person intersecting with the ray, the sensor counts the event as detected motion. If the person then remains continuously detected in the same detection zone after the initial detected motion, no further motion event is registered until the person either exits the current detection zone or is detected in any of the other detection zones.

The software used to control both the real and simulated version of the Panasonic PIR sensor in our study was developed by Sony. Due to the software being proprietary, details regarding the software logic of the sensors cannot be disclosed in this paper. A simplified description of the sensor logic, however, is that it measures movement roughly once per second and the output of each measured second is binary: either there was no motion detected, 0, or there was motion detected, 1. The sensor logic implemented in the Unity simulation is, for all intents and purposes identical, to the logic being used by the real-world sensors.

**Figure 2:**
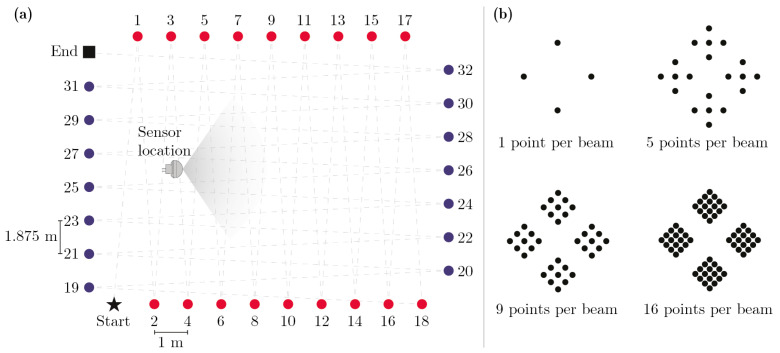
(**a**) rectangular area surrounded by nodes used to test the impact of altering the number of points per beam in the synthetic motion sensor; (**b**) arrangement of rays in the four different point densities evaluated.

To determine whether or not it would be beneficial to add more points per detection zone than five, a simple experiment was conducted. An empty rectangular 20 × 30 m space was created in Unity. 34 nodes were then positioned along the perimeter of the rectangular area as can be seen illustrated in the left part of Figure 2. A simulated humanoid, which is described in Section 2.2, was then instructed to walk in straight paths between each node from start to end in consecutive order. A synthetic motion sensor was placed inside the rectangle at a height of 3 m and a downward-facing tilt of 20° at the location indicated in Figure 2. As the humanoid walked in a zigzag pattern between each pair of nodes it crossed the sensor’s field of view in a multitude of incidence angles. The experiment was repeated four times, each time with a different number of ray-casted points per beam in the motion sensor. The number of points per beam tested was 1, 5, 9, and 16, resulting in 68, 340, 612, and 1088 total number of rays cast per sensor respectively. The arrangement of the points within the detection zones of the sensor is shown in the right part of Figure 2. During the first iteration of the experiment, when one point per beam was used, a total of 170 motion events were detected by the sensor. In all three other experiments, a total of 216 motion events were detected. This indicates that increasing the number of points per detection zone above five is likely unnecessary for detecting the crude body parts of our humanoid model. Decreasing the number of points per detection zone to one, however, could cause a substantial part of the humanoid movements to go undetected.

### 2.2. Humanoid Model

**Figure 3:**
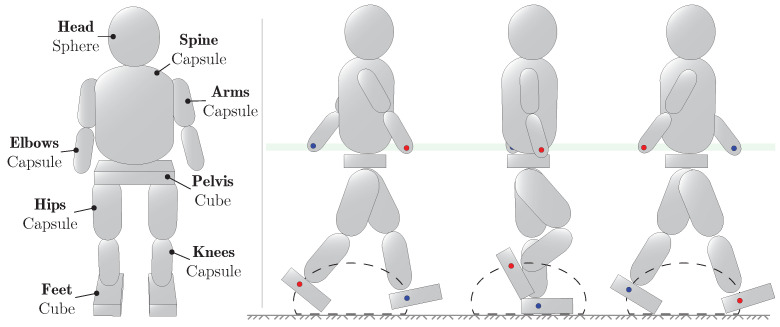
Front view of humanoid model (**left**) and illustration of concept used to perform locomotion by pushing the feet of the humanoid around in elliptical orbits (**right**).

A human-like body structure was generated in Unity by joining together 13 solid objects of primitive geometrical shapes: one sphere, three cubes, and nine capsules. Which body part each of the 13 primitive shapes corresponds to in the humanoid model can be seen illustrated in the left part of Figure 3. The dimensions of each body part were determined by using the anatomy of a 186 cm male as a reference. Since the humanoid model is composed of primitive geometrical objects whereas a real person is not, it is not straightforward to accurately map the measurements of a real person onto the body parts of the humanoid model. The body part dimensions used in the model are therefore crude approximations of the human anatomy.

Each of the 13 body parts was assigned a mass computed as a fraction of the total body mass which was set to 80 kg. What percentage of the total body mass each body part would be assigned was determined using the percentages from table 4 of Plagenhoef et al.’s study on anatomical data [25]. The primary purpose of assigning mass to the body parts of the humanoid is to enable the physics engine of the simulator to realistically apply gravitational forces to the model.

Relevant body parts such as head and spine, hips and knees, etc., were joined together using Unity’s character joints. Each joint was configured with three-dimensional angular restrictions approximated from the male reference person, thus restricting the way in which the humanoid’s body parts were allowed to move in relation to each other. As a result, any movement or force applied to one individual body part of the humanoid model causes all of its connected body parts to respond to the position change/force, leading to a cascade of motion in the body parts of the model. For instance, applying an upward force to one of the feet of the humanoid model would cause the knee connected to the foot to raise along with the foot. In turn, the hip that is connected to the knee would respond to the movement in the knee by changing its rotation and position, and so on. The cascade of motion caused by the manipulation of one body part is solved in real-time by the physics engine in Unity. One benefit of this is that rather complicated motion can be achieved with little programming effort.

To achieve human-like strides when moving the humanoid model in and out of rooms, only two regions of the body were manipulated, namely the feet and the elbows. Around each foot of the humanoid, an ellipse was constructed. The length of the ellipse corresponds to the stride length when walking and was set to 0.5 m. The height of the ellipse corresponds to the stride height of the humanoid’s walk and was set to 0.3 m. To emulate human-like strides a force was applied to each foot of the humanoid that would push the foot around the ellipse in a loop. The other parts of the legs—the knees and the hips—were not programmatically controlled, their movements during a stride were determined by the physics engine as a consequence of the forces applied to the feet. To ensure that the left leg moved forward as the right leg moved backward during locomotion, an offset was added between the starting points of the left and right ellipse. The stride frequency of the walk could then be adjusted by changing how fast the feet are pushed around the ellipses. In our experiments, the walking speed of the humanoid was set to 1 m/s. During a human walking cycle, the left arm commonly swings back and forth along with the right leg and vice versa. To incorporate this into the walk of our humanoid, a force was applied to the left elbow that pushed it towards the position of the right foot. Conversely, a force was applied to the right elbow that pushed it towards the left foot. The combined effect of adding forces to the feet and elbows of the model resulted in a relatively human-like walking style that can be seen illustrated in the right part of Figure 3.

All body parts of the humanoid are affected by gravity once Unity’s physics engine is started, i.e., if left to its own devices the humanoid model falls to the ground once the simulation is started. Balancing the humanoid to remain standing by continuously adjusting the body parts to counteract gravity is no trivial task. To circumvent the need for solving challenging balance problems, the spine position of the humanoid was frozen in the vertical direction during the simulations, i.e., the humanoid was simply prevented from ever falling due to gravity.

To allow the humanoid model to move from one point to another in the simulated world, the simulated space was discretized into a 0.1 × 0.1 m grid. Appropriate paths between two given tiles in the grid—paths that avoid obstacles and are not redundantly lengthy—were identified using an implementation of the A* pathfinding algorithm [26]. Given a starting position and a target position the humanoid was thus capable of finding an obstacle-free path to its target and could follow the identified path whilst taking human-like strides.

**Figure 4:**
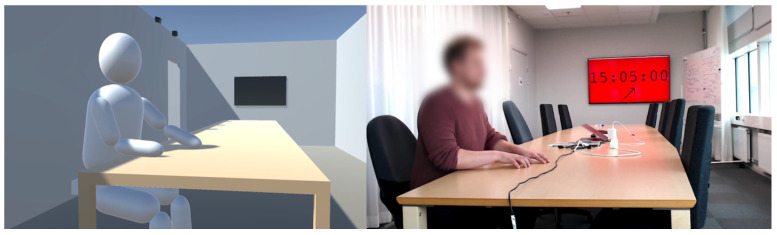
Snapshot taken from the simulated version of the experiment in Study A (**left**) and the real-world version of the same experiment (**right**). In both instances seat S1 is being occupied.

In addition to walking, the humanoid was also bestowed with the ability to sit down. Sitting down was achieved by temporarily freezing the rotation and position of the humanoid’s knees whilst releasing any motion restrictions in the spine—since the spine is prevented from changing position in the vertical direction when the humanoid is walking and standing. Once the vertical movement restrictions are released, the upper body will immediately start to lower due to gravitational forces, which in turn causes the hips to bend backward. Once the hips reach an angle greater than 80° the rotation of the hips was frozen to prevent the humanoid from falling further towards the ground. At the same time, the vertical position of the spine was again locked. In essence, when the humanoid transitions from standing to sitting, the humanoid falls in a controlled way and is restricted from collapsing further once a posture resembling a person sitting down is reached. The result of the sitting down procedure is that the humanoid appears to sit mid-air in an invisible chair which can be seen in the left part of Figure 4. To raise the humanoid to a standing position again after sitting down, the movement restrictions on the spine and knees are removed and an upward force is applied to the pelvis and spine of the model. Once the spine has reached its original vertical position the upward forces are turned off and the spine position is locked again to prevent the humanoid from falling.

### 2.3. Meeting Room

**Figure 5:**
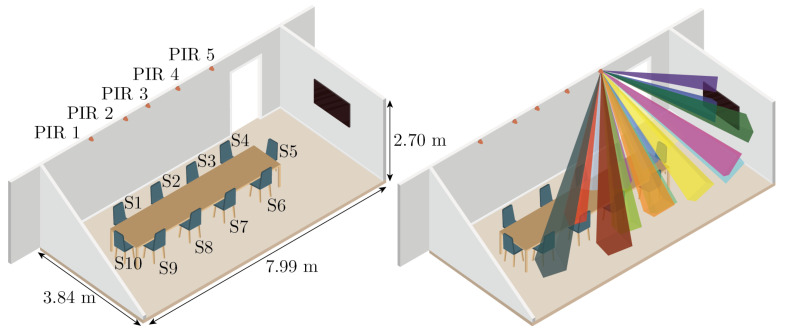
Illustration of the meeting room used during the experiments. The left part of the figure shows the naming convention used when referring to various sensors and seat positions in the room. The right part of the figure illustrates how the beams of one of the PIR sensors spread out into the room. PIR 1–5 indicates the respective position of each of the five passive infrared motion sensors in the room. S1–S10 represents the ten different seat positions in the room.

A 30.7 m² meeting room located in Sony’s office building in Lund, Sweden, was measured and then replicated in the Unity simulator. The meeting room was equipped with five PIR motion sensors of model Panasonic EKMC1604111 which were installed along the same wall in the room as the entrance door. Height-wise the sensors were positioned directly adjacent to the ceiling at a height of 2.65 m. All sensors were installed at a 20° downward tilting angle. The furniture of the meeting room consisted of a television and a rectangular table with room for ten seats. The geometry of the room and the position of the sensors and furniture can be seen illustrated in the left part of Figure 5. Aspects such as door position, sensor positions, and table placement were identical between the real-world version of the room and the Unity version of the room. The only difference between the real and simulated room was that the chairs were omitted in the simulated version of the room due to the humanoid model’s ability to sit in mid-air.

## 3. Experimental

### 3.1. Study A—Simulated vs. Real PIR Data

To enable direct comparison between our synthetically generated motion sensor data and motion sensor data collected in real life, two identical experiments were performed—one experiment taking place in the real room and one in the simulated version of the same room. The design of the experiment was as follows:

1. a person is initially positioned outside of the room of interest;
2. the person enters the room, walks to the first seat, and sits down;
3. the person then moves the arms and upper body in a predetermined way for three minutes;
4. after three minutes the person stands up and exits the room;
5. the experiment starts over from point 1) but this time the next seat in the room is chosen.

**Figure 6:**
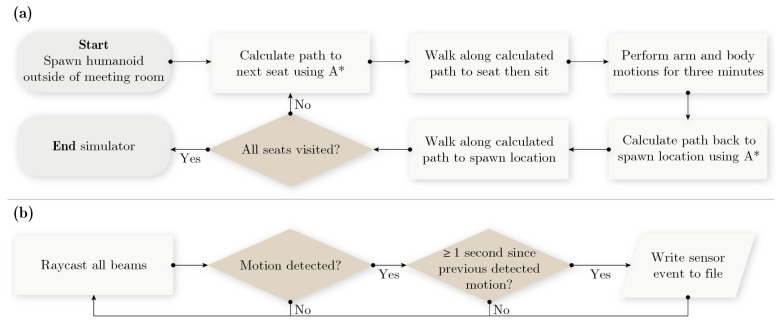
Flowcharts of the simulator developed for Study A. The upper flowchart, (**a**), describes the software logic of the humanoid. The lower flowchart, (**b**), describes the software logic of each individual motion sensor in the room. During the simulation both parts of the software are running simultaneously.

To ensure that the body movements performed during phase 3 of the experiment could easily be replicated in the simulator, the body movements were rather robotic in nature. The arms were placed on the table in front of the person and oscillated continuously ±10 cm back and forth with a frequency of approximately 1/3 Hz. I.e., one arm oscillation every third second. In the Unity implementation of the experiment the arm motion was realized by programmatically forcing the arms of the humanoid to follow two sine waves: 0.1×sin(2π/3×t) for the left arm and −0.1×sin(2π/3×t) for the right arm. The second type of movement performed during phase 3 of the experiment was a periodic tilt change of the upper body. The spine was either upright (0° tilt), forward-tilting at an angle of 15°, or backward tilting at an angle of −15°. Every 30 s during phase 3, a random number generator was used to determine which of the three tilt alternatives should be used. In the real-life version of the experiment, the random number generator dictating the upper body tilt was shown on the TV in the room, thus allowing the person conducting the experiment to receive real-time directions in his peripheral vision. Figure 4 shows a comparison between the simulated version of the experiment and the real-life version of the experiment. Figure 6 summarizes the developed simulator used in Study A in flowchart form.

Since the spine tilts were chosen randomly every 30 s in both the real-life version of the experiment and the simulated version, the spine adjustments are unlikely to be identical between the two versions of the experiment. In other words, the logic by which the spine motion was determined is identical between the experiments, but the outcome of the randomness can differ. To obtain motion sensor data from a range of different spine motion sequences the simulated version of the experiment was therefore repeated 200 times. Producing a distribution of different motion data outcomes for each simulated PIR sensor.

### 3.2. Study B—Sensor Sensitivity in Various Regions of the Room

Study B was designed to quantify how a PIR sensor’s ability to detect motion is influenced by the spatial position of the object/human being detected. Due to the nature of the experiment, the experiment in Study B was performed exclusively in the Unity simulator.

**Figure 7:**
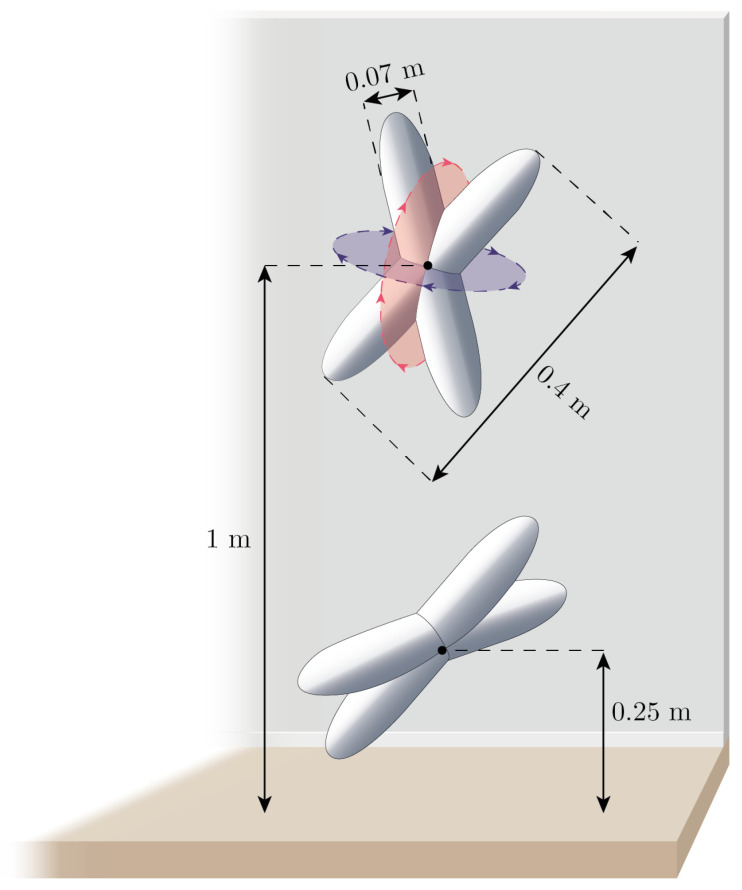
Illustration of spinning objects used to trigger the motion sensors in Study B.

Four capsule objects with a length of 40 cm and a width of 7 cm were pairwise grouped together to form two x-shaped objects which can be seen illustrated in Figure 7. The two x-shaped objects were positioned such that they levitated in mid-air, unaffected by gravity, at a height of 1 m from the ground and 0.25 m, respectively. The purpose of these objects was to cause as much easily reproducible movement as possible. To that end, the objects were programmed to continuously rotate along two directions as indicated in Figure 7 with one blue and one red rotation plane. The rotational speed was set to 100 degrees per second in both directions. The x-shaped objects were configured to be detectable by the PIR sensors in the simulation in the same way as the humanoid from Study A was.

The floor of the modeled room was then partitioned into a grid of 3 × 3 cm squares. The two x-shaped objects were then programmatically instructed to spin for exactly one minute in each of the approximately 34,000 squares. Thus giving each of the five PIR sensors in the simulated room the ability to detect the motion of the two spinning objects as a function of spatial location in the room. During the experiment in Study B, the table in the room was removed to ensure that all squares of the grid were free from obstructions.

## 4. Results

### 4.1. Study A

**Figure 8:**
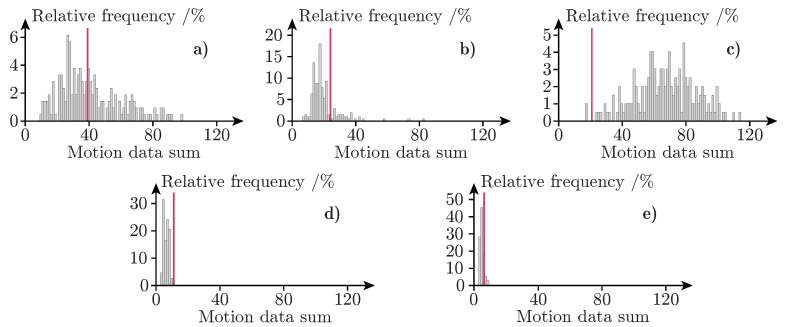
Relative frequency histograms of summed motion data for seat S1. Subplot (**a**) contains data from PIR sensor 1; (**b**) PIR sensor 2; (**c**) PIR sensor 3; (**d**) PIR sensor 4; (**e**) PIR sensor 5. Histogram data (gray bars) is from the simulated versions of the experiment. The red line in each subplot marks the result from the real-world version of the experiment for the same seat and sensor combination.

The simulated version of the experiment in Study A was repeated 200 times. Thus 200 different motion sensor time series were produced for each combination of sensor and seat. Since the experiment included five sensors and ten seats, a total of 10,000 (200 × 5 × 10) time series with motion sensor data was produced by the simulations of Study A. By calculating the sum of each time series, a single value is obtained for each of the 10,000 series. Each sum describes how many seconds during each of the 180 s long experiments a sensor detected motion. For each sensor and seat combination, there is a distribution of 200 sums that describe the total amount of motion registered during the experiment. Figure 8 shows the distribution of summed motion sensor data for every PIR sensor as the simulated experiment was conducted in seat S1. Provided that the simulator succeeds in accurately representing the real-world experiment, the motion sensor data collected in the real-life version of the experiment is expected to lie somewhere within the distribution of simulated motion data. The red lines in each subplot of Figure 8 indicate the sum of motion sensor data collected in the real-world version of the experiment. As can be seen in Figure 8, the simulated data for PIR sensors 1, 2, and 5 correspond well with the real-world sensor data collected as the experiment was performed in seat S1. The real-world sensor data from PIR sensor 3 is also within the range of the simulated data, but only by a narrow margin. PIR sensor 4 however, is outside the range of the simulated sensor data by a small margin.

Table 1 contains a summary of the real-world collected motion sensor data for each seat and sensor combination along with lowest, highest, and average summed motion sensor data for the 200 repetitions of the simulated experiment. In 34 of the 50 seat and sensor combinations, the sum of the real-world collected motion sensor data was within the lower and upper bounds of the simulated motion sensor data. The largest difference between simulation and the real world was observed in seat S7 sensor PIR 4 where the difference in collected motion sensor data was 52.

It is interesting to note that the real-world collected data contains large variations in detected amounts of motion between seats that are in close proximity to each other. For instance, PIR sensor 4 detected 128 motion events in seat S7, but the same sensor detected only 24 cases of motion in seat S8 which is a neighboring seat. Despite the fact that similar body movements were performed in both seats, 4.3x more motion was detected in seat S7 compared to S8. Interestingly, large variations in sensor output between neighboring seats were also found in the simulated data. The simulated version of PIR sensor 2 for instance, detected an average of 93 motion events in seat S7, but only an average of 6 motion events in seat S8. This phenomenon is examined further in Study B.

For 36 sensor and seat combinations, the average amount of simulated motion sensor data was lower than the real-world collected data. For 11 combinations the average amount of simulated motion sensor data was higher than the real-world sensor data. Provided that discrepancies between the humanoid model and the real person performing the experiment are not the cause of this, it could suggest that the simulated PIR sensors tend to under-report motion compared to the real-world sensors.

**Figure 9:**
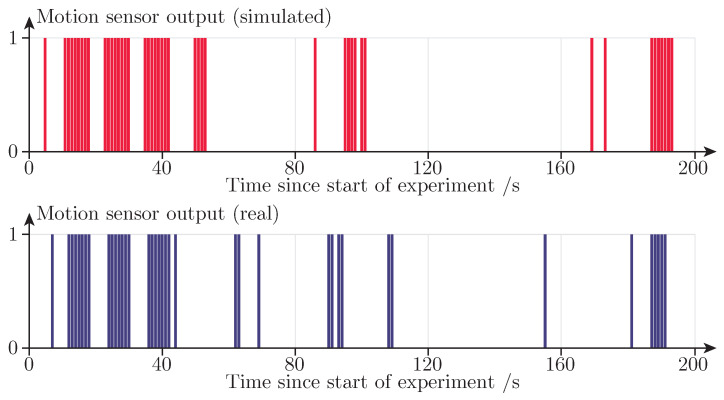
PIR sensor output as a function of time. The upper subplot shows simulated motion sensor data from one of the repetitions of the experiment in Study A. The lower subplot shows the motion sensor data collected during the real-world experiment. Both time series originate from sensor PIR 1 and seat S1. The sum of the simulated series is 45, whereas the sum of the series from the real-world sensor is 39.

Figure 9 shows a time series of raw sensor output from both the real and the simulated version of sensor PIR 1 whilst the experiment is performed in seat S1. The figure shows the sensor data as it exists before it has been summed into the integer values displayed in Table 1. As can be seen in Figure 9, the simulated sensor data is strikingly similar to the data collected in the real-world experiment for some seat and sensor combinations.

### 4.2. Study B

**Figure 10:**
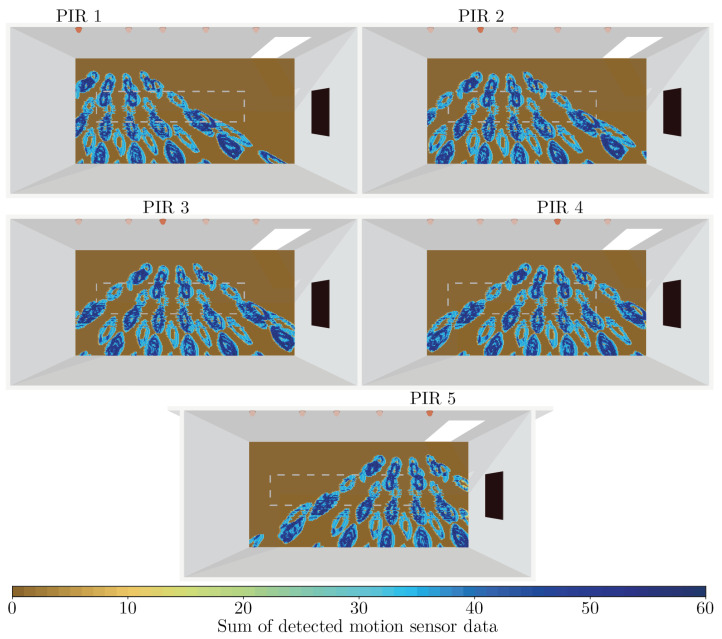
Heatmaps showing the sum of simulated motion sensor data caused by the spinning objects at various locations in the room. Each of the five subplots shows data registered by one of the five simulated PIR sensors. The white dashed rectangle indicates the position of the table in the room during Study A.

The motion sensor data detected in each square of the grid in the Study B experiment was summed into an integer value. The integer values of every square can be seen in Figure 10 arranged as a heatmap for each of the five PIR sensors. Since the PIR sensors detect movement no more than once per second and since the rotational movement of the objects in Study B was performed for 60 s in each square, the sum of the detected motion ranges from 0—meaning no motion was detected at all in the square—to 60.

As can be seen in Figure 10, how prone a PIR sensor is to detect the spinning objects’ motion varies greatly depending on where in the room the objects are spinning. Within a sensor’s field of view, multiple island-like formations of high detection ability appear. Between the areas of high sensor, sensitivity is areas of low to non-existent detection ability. Excluding squares where no motion at all was detected, the average difference in motion sensor sum between two neighboring squares was 8.8 across all PIR sensors. The most extreme difference observed between any two neighboring squares in the grid was 59. A difference of 59 between two neighboring squares implies that there are regions in the room where a spatial distance of just 3 cm determines if the sensor is unable to detect motion, or is highly susceptible to detecting motion.

## 5. Discussion

The simulated experiment of Study A was repeated 200 times. Since the random spine tilt of the humanoid could vary between the simulation repetitions some variability in sensor output is to be expected. However, for several seat and sensor combinations, we obtained a very large variability in the amount of motion data generated by the different repetitions. For instance, as can be seen in Table 1, the combination seat S8 and sensor PIR1 detected 5 motion events in one of the simulation iterations and 123 motion events in another. Furthermore, we also observed variations of a similar magnitude in the real-world motion sensor output when comparing the data from one PIR sensor for two neighboring seats, such as seats 7 and 8 for PIR sensor 4. If such large variations had been observed exclusively in the simulated data we would probably have assumed that the simulator is inaccurate. However, since surprisingly large variations in sensor output were also observed in the real-world sensors, we hypothesize that much of the variation is a consequence of the regions of high and low sensor sensitivity discovered in Study B. As can be seen in Figure 10, the sensitivity of a PIR sensor varies greatly depending on where in the room a motion is performed. Changing position by a few decimeters, such as when switching between neighboring seats, could thus be the difference between being located in one of the sensor’s blind spots and being located in an area of high detection sensitivity. Similarly, in the simulated experiment, the spine tilts of certain repetitions of the experiment could have caused the humanoid to lean predominantly in one direction, which could then have caused the humanoid to enter a region of high sensor sensitivity. Due to the stochastic nature of the simulation, other repetitions could instead predominantly lean in the opposite direction, causing the humanoid to enter a region of low sensor sensitivity.

It should be noted that the pattern of low and high PIR sensor sensitivity shown in Figure 10 is a result of simulating spinning objects of a certain shape with a certain rotation and a certain vertical height from the floor. Making changes to the spinning objects would likely also cause changes to the emerging pattern. In our sensors motion is defined as a change in beam activity. If the spinning objects would have been drastically smaller, such that they were small enough to remain within the field of view of a single beam of the sensor as they rotate, less motion would likely have been detected, particularly in the center of the beams. Most of the movement would then likely be detected at the edges of the beams, where the spinning objects can cross in and out from the beam as they rotate. This would thus likely change the island formations into atoll formations with a perimeter of high sensitivity and a center of low sensitivity. On the other hand, changing the vertical height at which the objects spin would change where in the room the objects intersect with the sensor beams. Which would then likely preserve the island-like formations but would offset where in the room the islands are formed. The island formations are seen in Figure 10 may therefore not be perfectly representative of the sensor sensitivity pattern one could expect when detecting objects of other shapes at other heights, such as when detecting real people sitting at a table. Furthermore, since the simulated PIR sensors detect motion using ray casting they have very sharply defined detection zones. The real-world PIR sensors operate differently; they rely on a lens to focus the influx of infrared radiation from a detection zone onto the sensor. It is therefore possible that the detection zone boundaries in the real-world sensor are fuzzier than the boundaries of the simulated sensors. If this is the case the sensor sensitivity pattern that is shown in Figure 10 for the simulated sensors will likely be blurrier and less well-defined for a real-world sensor.

When attempting to perform the experiment of Study A as similarly as possible both in the real room and the simulator, a few sources of potential inaccuracies should be mentioned. Among the most difficult things to accurately replicate was the position of the seats. Room dimensions and sensor positions were static throughout the experiment and could therefore reliably be measured. As a person sits down on a chair, however, the chair is first pulled backward from under the table and is then pushed forward again as the person sits. This means that even if the position of the seats were to be accurately measured before the real-world experiment, the chairs will slightly change position during the experiment as the person interacts with the chairs. Because of this, there may have been unintentional deviations in seat position between the simulated and real-world version of the Study A experiment. And as is evident from the results of Study B, small differences in position could cause a large difference in sensor output. Another source of inaccuracy comes from spine tilts. It is easy for the simulated humanoid to accurately rotate the spine to −15°, 0°, or 15° tilt, but when a real person attempts to do so the tilt is just estimated and will naturally deviate slightly from the intended target tilt. Furthermore, the humanoid we created navigates by partitioning the world into a grid of 10 × 10 cm squares and applying the A* pathfinding algorithm to the grid. This means that given a target position, such as a seat, the humanoid rounds the position to the nearest available 10 × 10 cm square, which often leads to a spatial deviation of a few centimeters.

## 6. Conclusions

This paper demonstrates the viability of using a simulator to generate synthetic PIR motion data. The motivation for why such a simulator is needed is that it can be used to produce both synthetic PIR motion sensor data and the accompanying occupancy ground truth data needed for solving occupancy estimation using supervised learning. The degree of authenticity in our produced synthetic sensor data was tested by performing a choreographed set of motions in ten locations of a real-world room equipped with five PIR sensors and then simulating the same set of conditions using the game engine. It was found that in 34 out of the 50 compared circumstances, the synthetically generated PIR data matched the real-world PIR data rather well. In 16 compared circumstances, the simulated data did not match reality. We hypothesize that the observed discrepancies between simulated and real-world PIR data are primarily due to two sources of error: (i) there is a fundamental difference in how the real-world sensors operate and how the simulated sensors detect motion. The lens of a real-world PIR sensor optically focuses infrared radiation towards the sensor. The sensors in our simulation, however, detect motion using ray casting. Ray casting causes the detection zones to be incredibly sharply defined, leading to situations where the sensor can transition from being highly capable of detecting motion performed in one location to being unable to detect motion performed in a location just a few centimeters away. It is reasonable to assume that the detection zones of the real-world sensors are less sharply defined, and that motion performed right at the edge of a detection zone has a probabilistic chance of triggering a motion event in the sensor or not. (ii) Some differences between the position of the human and the simulated humanoid during our experiment were unavoidable. Since the seats in the real-world room were not fixed to the floor, they moved slightly during the experiment which made it difficult to accurately determine what position the seats should have when replicating the room in the simulator.

The results of the performed simulations also suggest that the likelihood that a PIR sensor detects a motion depends greatly on the angle and distance to the person carrying out the motion. A relatively minor change in the position of a person can cause an observing PIR sensor’s output to increase or decrease by hundreds of percent. This finding illustrates why mapping PIR motion sensor data to occupancy count is a challenging problem. Due to the spatially complex and non-linear variability in PIR sensor output throughout a room, a large number of observations are likely needed for a supervised learning model to robustly learn the mapping between PIR sensor data and occupancy. This in turn further motivates why being able to synthetically generate large datasets of realistic PIR data is useful. The complex pattern also highlights why it could be beneficial for occupancy estimation purposes to equip rooms with other sensors in addition to a wall-mounted PIR sensors, such as motion sensors mounted in doorways.

As the experiments in this paper were limited to simulating simplistic body motions, future studies should investigate how to incorporate more natural body movements in a simulated humanoid that accurately mimics motions performed in real life during various activities. Additionally, future studies should examine if the level of realism offered by the simulated motion sensor data is enough to bridge the reality gap, such that supervised machine learning models can be trained on the synthetic data and generalize to motion sensor data originating from real-world PIR sensors.

## Figures and Tables

**Table 1:** Summary of motion sensor data collected during the experiments of study A for every combination of seat and motion sensor. ’real’ indicates the sum of motion sensor data collected during the real-world version of the experiment. min${}_{\mathrm{sim}}$, max${}_{\mathrm{sim}}$ and mean${}_{\mathrm{sim}}$ denotes the minimum, maximum and mean amount of motion sensor data collected across all repetitions of the simulated experiment. The ’✓’ symbol indicates that the motion sensor data collected in the real-world experiment is within the lower/upper range of the simulated motion sensor data. Conversely, the ’✗’ symbol indicates that the real motion sensor data is outside the bounds of the simulated data.

Sensor		PIR1	PIR2	PIR3	PIR4	PIR5
Seat						
S1	real	39	24	21	11	6
	min${}_{\mathrm{sim}}$	10	7	17	3	3
	max${}_{\mathrm{sim}}$	98	83	114	10	9
	mean${}_{\mathrm{sim}}$	41	20	67	6	5
		✓	✓	✓	✗	✓
S2	real	27	22	28	8	7
	min${}_{\mathrm{sim}}$	7	6	7	3	2
	max${}_{\mathrm{sim}}$	116	48	33	9	7
	mean${}_{\mathrm{sim}}$	46	17	13	6	3
		✓	✓	✓	✓	✓
S3	real	21	28	35	27	12
	min${}_{\mathrm{sim}}$	3	3	4	9	3
	max${}_{\mathrm{sim}}$	5	7	12	63	9
	mean${}_{\mathrm{sim}}$	4	5	7	17	5
		✗	✗	✗	✓	✗
S4	real	5	17	20	25	25
	min${}_{\mathrm{sim}}$	0	2	3	3	7
	max${}_{\mathrm{sim}}$	30	5	38	8	69
	mean${}_{\mathrm{sim}}$	4	4	5	5	18
S5	real	0	0	15	20	49
	min${}_{\mathrm{sim}}$	0	0	3	8	3
	max${}_{\mathrm{sim}}$	0	0	23	59	37
	mean${}_{\mathrm{sim}}$	0	0	7	17	7
		✓	✓	✓	✓	✗
S6	real	0	0	14	81	38
	min${}_{\mathrm{sim}}$	0	0	3	3	6
	max${}_{\mathrm{sim}}$	0	1	66	59	42
	mean${}_{\mathrm{sim}}$	0	0	14	15	13
		✓	✓	✓	✗	✓
S7	real	17	22	86	128	89
	min${}_{\mathrm{sim}}$	2	39	7	6	11
	max${}_{\mathrm{sim}}$	30	118	75	76	93
	mean${}_{\mathrm{sim}}$	4	93	13	11	28
		✓	✗	✗	✗	✓
S8	real	22	27	19	24	77
	min${}_{\mathrm{sim}}$	5	3	9	4	7
	max${}_{\mathrm{sim}}$	123	10	95	24	56
	mean${}_{\mathrm{sim}}$	66	6	49	9	16
		✓	✗	✓	✓	✗
S9	real	23	37	20	51	24
	min${}_{\mathrm{sim}}$	3	28	4	9	19
	max${}_{\mathrm{sim}}$	10	94	48	60	100
	mean${}_{\mathrm{sim}}$	7	58	10	16	49
		✗	✓	✓	✓	✓
S10	real	37	80	80	31	35
	min${}_{\mathrm{sim}}$	12	12	13	21	11
	max${}_{\mathrm{sim}}$	100	95	91	112	32
	mean${}_{\mathrm{sim}}$	31	30	36	57	21
		✓	✓	✓	✓	✗
